# Effects of memory and attention on the association between video game addiction and cognitive/learning skills in children: mediational analysis

**DOI:** 10.1186/s40359-024-01849-9

**Published:** 2024-06-24

**Authors:** Amani Ali Kappi, Rania Rabie El-Etreby, Ghada Gamal Badawy, Gawhara Ebrahem, Warda El Shahat Hamed

**Affiliations:** 1 Department of Nursing, College of Nursing, Jazan University, Jazan, Kingdom of Saudi Arabia; 2 Psychiatric and Mental Health Nursing Department, College of Nursing, Mansoura University, Mansoura, Egypt; 3 Pediatric Nursing Department, College of Nursing, Mansoura University, Mansoura, Egypt

**Keywords:** Child memory, Attention, Video game addiction, Learning skills, Children

## Abstract

**Background:**

Video games have become a prevalent source of entertainment, especially among children. Furthermore, the amount of time spent playing video games has grown dramatically. The purpose of this research was to examine the mediation effects of attention and child memory on the relationship between video games addiction and cognitive and learning abilities in Egyptian children.

**Methods:**

A cross-sectional research design was used in the current study in two schools affiliated with Dakahlia District, Egypt. The study included 169 children aged 9 to 13 who met the inclusion criteria, and their mothers provided the questionnaire responses. The data collection methods were performed over approximately four months from February to May. Data were collected using different tools: Socio-demographic Interview, Game Addiction Scale for Children (GASC), Children’s Memory Questionnaire (CMQ), Clinical Attention Problems Scale, Learning, Executive, and Attention Functioning (LEAF) Scale.

**Results:**

There was a significant indirect effect of video game addiction on cognitive and learning skills through attention, but not child memory. Video game addiction has a significant impact on children’s attention and memory. Both attention and memory have a significant impact on a child’s cognitive and learning skills.

**Conclusions:**

These results revealed the significant effect of video game addiction on cognitive and learning abilities in the presence of mediators. It also suggested that attention-focused therapies might play an important role in minimizing the harmful effects of video game addiction on cognitive and learning abilities.

## Introduction

The use of video games has increased significantly in recent years. Historically, such games are used more often by children. Despite the positive impacts of video games on socialization and enjoyment, empirical and clinical research has consistently demonstrated that many children can become addicted due to excessive use. Among Arab children and adolescents, the prevalence of video game addiction is 62% of 393 adolescents in Saudi Arabia, 5% in Jordan, 6% in Syria, and 7.8% in Kuwait [1, 2]. The varying incidence rates can be attributable to variations in the research population, cultural determinants, and evaluation or diagnostic standards.

In addition, video games, the internet, and other new technologies have become children’s top leisure pursuits. Today, they comprise a virtual environment in which thousands of gamers simultaneously participate worldwide; rather than being a personal or lonely leisure activity, they are often a group activity that establishes new social networks [3]. Although playing video games in moderation can have many positive effects, their exploitation may lead to addictions and societal issues [4]. The Diagnostic and Statistical Manual of Mental Disorders, Fifth Edition (DSM-5), identifies repetitive and persistent behavior related to online video games as the core element of addiction. This behavior should persist for at least 12 months and result in significant impairment. Additionally, addiction should be accompanied by psychological and social symptoms, as well as tolerance and withdrawal symptoms [5].

Different studies have examined the impact of video games on children’s cognitive abilities and school performance [6, 7]. The recent literature has shown how video games affect the brain and alter its functioning while being played. It demonstrates how specific cortical and subcortical structures are involved [8–10]. Research indicates that excessive play of the same typees of games might negatively impact school-age children’s cognitive and academic skills as well as their capacity to maintain and enhance memories [7]. Possible consequences of video game addiction may include memory and attention-related difficulties [4, 6, 11]. For instance, children’s memory scores negatively correlated with greater levels of video game addiction in Lebanon [6]. Furthermore, studies show that action-game players are more likely to succeed at short-term concentration tests while they perform below average in long-term, less exciting activities. At the point of game addiction, difficulties with focus are likely to become much more apparent [12]. Studies show a substantial association between gaming addiction and inattention, even after controlling other variables such as personality factors, anxiety and depression symptoms, and attention deficit hyperactivity disorder [13, 14].

Prior studies have illustrated the association between video game addiction and psychiatric disorders, social phobia, mental well-being, and risky health behaviors [15–17]. Another study shows an association between video game addiction and memory, attention, cognitive, and learning abilities among Lebanese children [18]. However, all of these studies explain the association without controlling for any history of mental or behavioral disorders such as ADHD, anxiety, or depression. However, to the best of our knowledge, a few studies have specifically investigated the effect of attention and child memory on the relationship between video game addiction and cognitive and learning abilities in Egyptian children. Therefore, this study aimed to explore the mediation effect of attention and child memory on the association between addiction to video game and cognitive and learning abilities among Egyptian children. Our hypotheses were: (1) child attention mediates the relationship between video game addiction and cognitive and learning abilities among Egyptian children; and (2) child memory mediates the relationship between video game addiction and cognitive and learning abilities among Egyptian children.

## Literature review

Video games have transformed into complex experiences that embody principles recognized by psychologists, neuroscientists, and educators as crucial for behavior, learning, and cognitive functions. While video games offer social and entertainment benefits, extensive research indicates that their excessive use can lead to adverse psychological consequences and even addiction in a minority of players. Symptoms like impaired control over gaming and prioritizing games over daily responsibilities may signify gaming addiction [19].

The Diagnostic and Statistical Manual of Mental Disorders (DSM-5) acknowledged video game addiction as an internet gaming disorder in its fifth edition, highlighting the need for further research [20]. Similarly, the 11th edition of the International Classification of Diseases (ICD-11) classified gaming disorder as a recurrent pattern of gaming behavior that encompasses both online and offline gaming [21]. Scientific evidence indicates that addictions can develop due to a combination of genetic susceptibility and repeated exposure to specific stimuli [22].

Growing public concerns have emerged regarding the potential negative impacts of video games, notably on children’s memory [23]. Individuals with various behavioral disorders and those with addictive tendencies often find their memory, crucial for comprehension and cognitive abilities like memory updating and working memory, compromised [24]. Although some research delves into video games’ effects on cognitive functions and academic achievement in children [25, 26], the impact on memory remains a contentious topic.

Despite being a leisure activity, video gaming can pose issues for certain children, impacting their ability to focus. Meta-analysis and systematic reviews by Ho et al. and Carli et al. indicated a link between inattention and addiction to the internet and gaming [27]. Additionally, numerous studies corroborated this connection, demonstrating a robust correlation between the severity of inattention in ADHD and addiction to the internet or gaming. This correlation persisted even after controlling for factors such as depression and anxiety symptoms, as well as personality traits [27].

## Methods

### Study design and sample

This study has a cross-sectional descriptive design. It was conducted in two convienient selected preparatory schools, Emam Mohamed Abdo Preparatory and Omar Ibn Elkhatab Preparatory School. The two schools are affiliated with xxx. The participants were selected at random from the list of school principals. The research was open to all students between the ages of 9 and 13 with no history of physical, mental, or cognitive disorders. Each student’s parents provided the questionnaire responses. Using the G-power software 3.1.9.2, the study’s sample size was determined. Based on an average effect size of f = 0.15, a 2-sides test at alpha = 0.05, a statistical power (1-β) of 0.95, and eight predictors (age, gender, educational level of the child and mother, video game addiction, memory, attention, and learning abilities), power analysis was performed. A minimum of 166 participants were required based on these criteria.

### Ethical consideration

The study approved by the Research Ethics Committee (REC) of Mansoura University’s Faculty of Nursing (IRB P0506/9/8/2023). The study’s purpose, methodology, duration, and benefits were also explained to the directors of the two selected institutions. Mothers’ consents obtained after explaining the study’s objective and the data kept confidential. The participants were informed that they had their right to withdraw from the study at any time.

### Data Collection

The following tools were utilized in the study:

#### Socio-demographic questionnaire

Child and mother’s information was collected, such as age, sex, number of children, and level of education.

#### Video game addiction

We used the Game Addiction Scale for Children (GASC) to measure children’s video game addiction. The GASC developed by Yılmaz, Griffiths [28] according to DSM criteria to evaluate gaming addiction. It includes 21 self-reported items rated on five-point Likert scale (from 1 = never to 5 = very frequently), where higher score shows more hazardous online gaming usage. An individual’s total score can range from a minimum of 21 to a maximum of 105; a score above 90 may be a sign of a video game addiction. It is also emphasized that this is not a diagnostic tool, however, but merely an indicator that a child may have a gaming addiction. Such a diagnosis could only be made by a comprehensive clinical evaluation. Seven criteria for video game addiction are determined by the scale: salience, tolerance, mood modification, withdrawal, relapse, conflict, and issues. The scale shows an acceptable internal consistency reliability (*r* = 0.89, *p* < 0.001) [19].

#### Children’s memory

We used the Children’s Memory questionnaire (CMQ) to assess children’s memory rated by their parents. The CMQ developed by Drysdale, Shores [29]. It included 34 items that rated on a five-point Likert scale ranging from 1 = never or almost never, to 5 = more than once a day. Higher scores indicate a more significant reduction in the cognitive domain. The scale is divided into three subscales: working memory and attention, visual memory, and episodic memory. The Cronbach alpha value for the episodic memory subscale was 0.88, the visual memory is 0.77, and the working memory is 0.84 [29].

#### Attention of children

The Clinical Attention Problems Scale was used to measure children’s attention level in the morning and afternoon. This scale was developed by Edelbrock and Rancurello [30] and includes 12 items. The possible responses are 0 = not true, 1 = somewhat or sometimes true, and 2 = very often or often true. The higher the scores, the more attention there is. The Cronbach alpha values for the clinical attention problem in the morning is 0.84 and for the afternoon is 0.83.

#### Cognitive and learning skills

We used the Learning, Executive, and Attention Functioning (LEAF) scale to measure children’s cognitive and learning skills. The LEAF scale is a self-reported 55 items scale developed by Castellanos, Kronenberger [31]. The scale assesses core cognitive abilities and related academic and learning abilities. The LEAF assesses cognitive skills such as attention, processing speed, working memory, sustained sequential processing to accomplish goals (such as planning and carrying out goal-directed tasks), and new problem-solving. Moreover, the LEAF approach takes into account academic functioning, declarative/factual memory, and understanding and concept formulation.

The LEAF includes 55 items, with 11 academic subscales that rate a person’s reading, writing, and mathematics proficiency. The LEAF is divided into subscales that measure comprehension and conceptual learning, factual memory, attention, processing speed, visual-spatial organization, sustained sequential processing, working memory, new problem-solving, mathematics, basic reading, and written expression skills. Each subscale has the same number of items. The responses were rated on a three-point scale ranging from 0 to 3. Higher scores indicate more significant issues with cognition. The five component items are added to provide the subscale score for each of the 11 subject areas. Three criterion-referenced ranges are established for the interpretation of LEAF subscale raw scores. Out of nine, a score of five to nine is classified as the “borderline problem range,” a score of less than five as the “no problem range,” and a score of nine or above as the “problem range.” The Cronbach alpha value for the LEAF scale is 0.96.

### Validity and reliability

Study tools were translated into Arabic by the researchers. Five pediatric nursing and psychiatric and mental health nursing experts tested them for content validity. At first, the scales were translated into Arabic using a forward and backward translation method. The translated questionnaires were then adapted to fit Arabic cultural norms. Two highly proficient native Arabic speakers who are accomplished academics in the fields of psychiatry and mental health nursing, and hold the academic status of Full Professor translated the questionnaire from English to Arabic. An English-language expert who is fluent in Arabic back translated the Arabic version. Native Arabic speakers who were not involved in the translation process verified the final translation. The forward-to-back translation process was repeated until the comparative findings matched exactly. The questionnaires were then given to three Arabic psychiatric nursing professionals, who provided their opinions on its importance, relevance, and simplicity. The tools’ reliability was tested using Cronbach’s alpha test (tool I α = 0.86, tool II α = 0.81, tool III α = 0.95, and tool IV α = 0.95, respectively). Additionally, a confirmatory factor analysis were carried out to validate the content of the four scales after translation. The data collection methods were performed over approximately four months from February to May. Also, a pilot study was conducted to assess the study tools’ feasibility and determine the time required to complete the tools. 10% of the initial participants were randomly selected from the same schools. Minimal modifications were then made to the tools. Mothers of students who participated in the pilot study were excluded from the primary study. The data was collected for four months (February to May). An online Google form was created to collect data. The link was then shared with selected student parents through WhatsApp groups. The link outlined the study’s purpose and methods, and participants signed a consent form.

### Data collection procedure

We obtained permission to translate the study scales into Arabic. We collected data from February to May using an online Google Form for four months. The Google Form included full details regarding the study’s aims and processes to ensure transparency and establish participants’ trust. An extensive description of the response process additionally supports the Attention Problems Scale. For instance, mothers are required to respond to the items and their relevance to their children in the morning and afternoon. We distributed the survey link to the selected students’ mothers through WhatsApp groups as it was convenient and widespread among the target demographic. Before proceeding to the survey questions, participants were required to read and sign this consent form to ensure that participants received information about the study and voluntarily consented.

### Statistical analysis

We employed the Statistical Package for Social Science version 26 [23] to analyze the data. We analyzed the demographic data using descriptive statistics such as means, standard deviations, frequency, and percentages. In order to evaluate the mediator effects of memory and attention on the relationship between cognitive, academic, and learning skills and video gaming addiction, we ran the multiple regression PROCESS macro with 5,000 bootstraps in SPSS version 3.4 [24]. We also included confounding variables, such as the age of the child, gender, the age of the mother, education, and job status, as covariates in the mediation model.

## Results

### Sample characteristics

There were 169 children their mothers responded to the study surveys. The children’s mean age was 13 (SD = 3.9), while the mothers’ mean age was 41 (SD = 7.1). According to mothers, the children were ranked third in their household. Most mothers (72%) said they lived in rural areas. About 61% of the families had at least three children. Half of the mothers had high school or less education, and more than half were unemployed. Most children were in middle school (72%), see Table 1.

**Table 1:** Demographic characteristics for children and mothers

Variables	(*N* = 169) Mean ± SD or *n* (%)
Child gender	
Boy	84 (49.7%)
Girl	85 (50%)
Child age	13 ± 3.9
Child education level	
Middle school or less	122 (72%)
High school	47 (28%)
Child order in the family	
1	36 (21%)
2	41 (24%)
3	61 (36%)
4 or more	31 (18%)
Mother’s age	41 ± 7.1
Mother education level	
High school or less	89 (53%)
Undergraduate	51 (30%)
Graduate	29 (17%)
Mother employment	
Yes	69 (41%)
No	100 (59%)
Number of children in the family	
Three or less	108 (64%)
More than three	59 (35%)
Residence	
Rural area	122 (72%)
Urban area	47 (28%)

### Study variables description

The mean scores for all scales are presented in Table 2. The mean score of the video gaming addiction total scale was 61 ± 19.3, indicating a moderate level of addiction. The attention total scale mean was 9 ± 6.50, indicating moderate attention problems. The mean score on the total scale for child memory was 80 ± 31,4, indicating moderate memory issues. Eight subscales of the LEAF had mean scores of 5: factual memory, processing speed, visual-spatial organization, sustained sequential processing, working memory, novel problem-solving, mathematics skills, and written expression skills. These mean scores indicate that a borderline problem exists. However, the mean scores for the comprehension and conceptual learning subscale, attention subscale, and basic reading skills subscale were below five, indicating that there was no problem.

**Table 2:** Study’s main variables description

Variables	Mean ± SD	Median	Minimum & Maximum
Video gaming addiction total score	61 ± 19.3	63	21–105
Self-control subscale	20 ± 7.1	20	7–35
Reword	31 ± 9.8	31	10–50
Involvement subscale	10 ± 3.9	9	4–20
Attention problem total score	9 ± 6.50	11	0–24
Child memory total score	80 ± 31.4	84	1-170
Working memory and attention subscale	24 ± 9.7	23	10–50
Visual memory subscale	22 ± 9.4	20	10–50
Episodic memory subscale	34 ± 13.3	33	14–70
LEAF total score	52 ± 34.8	53	0–151
Comprehension and conceptual learning subscale	4 ± 3.3	4	0–15
Factual memory subscale	5 ± 3.6	5	0–15
Attention subscale	4 ± 3.2	4	0–15
Processing speed subscale	5 ± 3.8	5	0–15
Visual-spatial organization subscale	5 ± 3.9	5	0–15
Sustained sequential processing subscale	5 ± 3.7	5	0–15
Working memory subscale	5 ± 3.8	5	0–15
Novel problem-solving	5 ± 3.8	5	0–15
Mathematics skills subscale	5 ± 3.8	5	0–15
Basic reading skills subscale	4 ± 3.3	4	0–15
Written expression skills subscale	5 ± 3.4	5	0–15

NOTE: LEAF, Learning, Executive, and Attention Functioning

### Mediating effect of memory, attention problem on the association between video gaming addiction and cognitive, learning, and academic skills

**Fig. 1:**
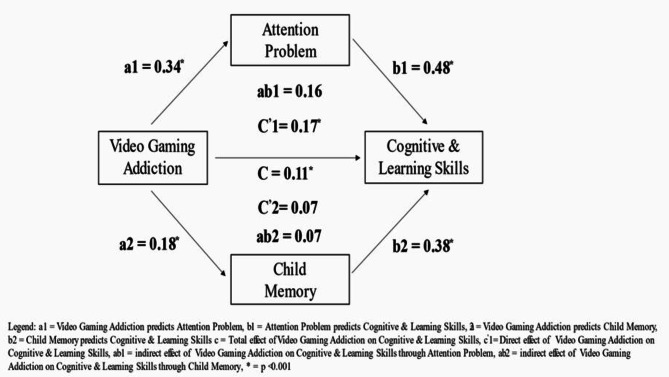
Mediation effect of attention problem and child memory on the association between video gaming addiction and cognitive and learning skills

Video game addiction had a significant impact on attention problems (b = 0.34, *p* < 0.001; a1), and child memory (b = 0.18, *p* < 0.001; a2). In turn, both attention problems (b = 0.48, *p* < 0.001; b1) and child memory (b = 0.38, *p* < 0.001; b2) had significant impact on cognitive and learning skills. The results reveal a significant indirect effect of video game addiction on cognitive and learning skills through attention problems (b = 0.17, CI: 0.82, 0.25; c^’^1). However, there was no significant indirect effect of video game addiction on cognitive and learning skills through child memory (b = 0.07, CI: -0.01, 0.16; c^’^2). The analysis revealed that confounding variables had no significant effect on the direct or indirect pathways linking video game addiction to cognitive and learning skills. The direct effect of video game addiction on cognitive and learning skills in the presence of the mediators was also found to be significant (b = 0.11, CI: 0.008, 0.401; c^’^-c). Figure 1 displays the mediation analysis findings.

## Discussion

Previous research has explored the relationship between video game addiction, attention, and memory. Some studies have focused on the relationship between video game addiction and cognitive and learning skills. Others have examined the association between video gaming addiction and all other variables (attention, memory, learning, and cognitive skills). However, no study has explicitly examined the direct and indirect effect of video gaming addiction on learning and cognitive skills through the mediation effect of attention and memory.

This study was done on a sample of Egyptian school children to evaluate the mediation effect of attention and memory on the relationship between video game addiction and cognitive and learning abilities in children. The present study reveals that a gaming addiction can significantly impact attention and memory. This result agrees with Farchakh, Haddad [6], who conducted a study on a group of Lebanese school children aged 9 to 13 to investigate the association between gaming addiction, attention, memory, cognitive, and learning skills. They found that a greater degree of addiction to video gaming was significantly associated with worse attention scores and worse memory scores. An earlier study suggests that the link between inattention and video game addiction could be described by game genres’ immediate response and reward system. Alrahili, Alreefi [2] suggest that this may alleviate the boredom typically reported by inattentive users while simultaneously introducing a lack of responsiveness to real-world rewards. Another study on Turkish schoolchildren aged 10 to 16 years old revealed that the total recall scores of the subject group (children who regularly play video games) are significantly lower than those of the control group (children who do not regularly play video games; [7]).

The current study demonstrates that attention and child memory significantly impacted cognitive and learning skills. This agrees with the opinion of, Gallen, Anguera [32], who argues that children and young people process information differently, affecting the performance of various cognitive tasks. Additionally, this result disagrees with the findings of Ellah, Achor, and Enemarie [26], who have stated that students’ working memory has no statistically significant correlation with learning and problem-solving skills. Moreover, their same study showed that different measures of working memory can be attributed to a small variation in low-ability students’ problem-solving skills.

The results revealed a significant indirect effect of video game addiction on cognitive and learning skills through attention. This could be related to the relationship between attention and learning skills. Attention is an essential factor in the learning process because it helps a person make efficient use of data by directing their learning to relevant components and relationships in the input material. If a student can pay attention, they may be able to better retain and understand this material; if not, a lack of attention may lead to difficulties in learning and academic performance. As video gaming addiction affects students’ attention, it may directly affect learning skills [33]. Another study agrees with the current result, revealing that video game addiction negatively affects adolescents’ learning skills and grade point average [34].

A child’s memory has an effect on their cognitive and learning skills. Encoding, consolidating, and retrieving experiences and information are the foundation for learning new skills and knowledge [35]. Video game addiction affects children’s memory. Hence, the expectation is that video game addiction directly affects cognitive and learning skills. However, the present study reveals no significant indirect effect of video game addiction on cognitive and learning skills through child memory. For example, perceptual attention to the exterior world and reflective attention to interior memories need modification of shared representational components in the occipitotemporal cortex. This is shown in episodic memory by recovering an experience from memory, which includes reactivating some of the same sensory areas used during encoding. Furthermore, the prefrontal cortex involves continuous and reflecting attention [36]. The prefrontal cortex controls memory recall by choosing target memories and filtering or suppressing competing memories [36].

Another aspect that may be responsible for the absence of a mediating effect of memory on the association between video game addiction and cognitive and learning skills is the presence of the many factors that affect learning and cognitive skills besides memory alone. Life circumstances can affect learning skills rather than memory itself, for example. Problem solving (one of the learning skills) requires a brain that works effectively. Therefore, it is critical to address needs such as physical health, which is influenced by self-care needs such as diet, sleep, and relaxation, as well as children’s social and emotional needs. Furthermore, learning experiences that use all the senses, rather than only hearing or seeing information, result in effective and straightforward information retrieval from memory during problem-solving processes. Such abilities are supposed to be acquired by active participation in learning activities by children [37]. Finally, long-term focus on online gaming may eventually lead to neglect in learning, leading to a deterioration in learning performance [38].

### Limitations

Our study has some limitations. First, we administered the Clinical Attention Problems Scale only once per student rather than conducting repeated measurements in the morning and afternoon. This approach overlooks potential daytime variations in attention levels, limiting our understanding of each child’s attentional profile. This choice was driven by practical considerations such as reducing the testing burden and participant fatigue. Future research could address this limitation by implementing repeated assessments to comprehend better daytime patterns in children’s attention levels and their implications for learning and behavior. Causality analysis was not possible due to the use of a cross-sectional sample. In addition, some results may be attributable to the small sample size. To fully understand the complex interplay between video game addiction and cognitive outcomes, longitudinal studies and controlled experiments are necessary to provide more conclusive insights into the relationship. It was difficult to include both parents in the study, as most of the fathers said they were too busy to participate. Hence, mothers were the subjects of the study. Certain differences (or lack thereof) are probably artifacts of the sample size. As a result, our findings must be validated by analyzing larger samples. Despite these limitations, this work has the potential to provide insights and open new research avenues.

### Implications

Healthcare professionals should be aware of how much children participate in these games and be willing to engage in in-depth conversations with parents about the impact these games may have on children’s health. Therefore, periodical workshops should be held by pediatric and community mental health nurses to enhance student awareness of the effects of video games on their memory, attention, and academic performance. In addition, teaching programs should be held at schools to improve students’ attention, memory, learning, and cognitive skills.

## Conclusion

Video game addiction has a significant impact on children’s attention and memory. Both attention and memory have a significant impact on a child’s cognitive and learning skills. These results reveal a significant indirect effect of video game addiction on cognitive and learning skills through attention. However, video game addiction had no significant indirect effect on cognitive and learning skills through child memory. In the presence of the mediators, the direct impact of video game addiction on cognitive and learning skills was also significant.

## Data Availability

No datasets were generated or analysed during the current study.
